# Supplementary material for: Parasitic Nematode and Protozoa Status of Working Sheepdogs on the North Island of New Zealand
Source: Animals (Basel). 2019 Mar 18;9(3):94. doi: 10.3390/ani9030094 (PMC6466095; doi:10.3390/ani9030094)
Supplement: Supplementary file 1 [file animals-09-00094-s001.pdf]

Appendix A: Owner and Animal Details

Owners name……………………………………………………Dogs name……………………………………..

Section 1) Owner and Animal Details

*****Please complete as much of this section as you are able to*****

Owner……

Address……………………………………………………………………………………………………….

………………………………………………………………………………………………

………………………………………………………………………………………………

Contact Phone(s)……………………………………………………………………………

Email………

Animal-Name………………………………………………………………………..

Breed………………………………………………………………………………………………………..

Colour…………………………………………………………………………………………

Age …..…………….. **Sex:** Male / Female **Entire:** Yes☐ No☐

Pedigree

(If your dog has trial dog pedigree please record the details here. If your dog has no trial dog pedigree, or it is unknown, please leave this blank)

Sire……………………………………………………………………………………………

Dam……………………………………………………………………………………………

Other Details

………………………………………………………………………………………………

………………………………………………………………………………………………

………………………………………………………………………………………………

………………………………………………………………………………………………

………………………………………………………………………………………………

Record Number…………………..Date……………………….

Appendix B: Clinical Examination

Owners name…………………………………………………….Dogs name……………………………………..

Section 2) Physical Exam and Ophthalmic Exam

a) History / owners concerns

……………………………………………………………………………………………

……………………………………………………………………………………………

……………………………………………………………………………………………

……………………………………………………………………………………………

a) Previous eye exam? No☐ Yes☐ *(If yes record details on the back of the sheet)*

b) Physical Exam Checklist

	Normal	Abnormal
Distance Exam	☐	☐
Cardiovascular	☐	☐
Respiratory	☐	☐
Gastrointestinal	☐	☐
Genitourinary	☐	☐
Integument	☐	☐
Musculoskeletal	☐	☐
Ears	☐	☐
Lymph Nodes	☐	☐
Nervous System	☐	☐

Right **Left**

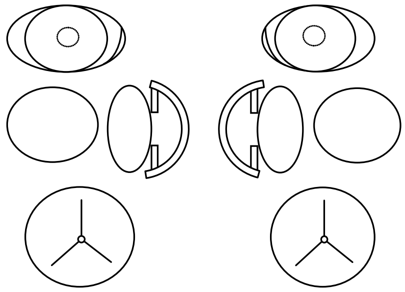

BCS __________

c) Eye Reflexes

	R.E.	L.E.
Menace	☐	☐
Dazzle	☐	☐
Palpebral	☐	☐

d) Parts of the eye

	Normal	Abnormal	L/R
Orbit	☐	☐	…..
Adnexa	☐	☐	…..
Cornea	☐	☐	…..
Iris	☐	☐	…..
Lens	☐	☐	…..
Vitreous	☐	☐	…..
Fundus	☐	☐	…..

c) Record details of abnormal findings below;

……………………………………………………………………………………………

……………………………………………………………………………………………

……………………………………………………………………………………………

……………………………………………………………………………………………

……………………………………………………………………………………………

……………………………………………………………………………………………

……………………………………………………………………………………………

d) Samples

		Record number on sample?
Faecal Sample	☐	☐
Blood Sample	☐	☐
Eye Exam	☐	☐

Record Number…………………….Date……………………….

Appendix C: Owner Questionnaire

OWNERS NAME____
DOGS NAME ____

SECTION 3) Owner Questionnaire

Please complete as much of this section as you are able to

IF THE ANSWER FOR A QUESTION DIFFERS BETWEEN YOUR DOGS LEAVE THE QUESTION BLANK AND I WILL WRITE DOWN THE DIFFERENCES BETWEEN THE DOGS WHEN I SEE YOU

1. **Is your dog(s) vaccinated?**

☐ Yes ☐ No ☐ Don't know

***If* Yes to (1); How often is your dog(s) vaccinated?**

☐ Only when a pup ☐ Annually ☐ Every 2 years
☐ Sporadically

2. **How often do you worm your dog(s)?**

☐ Only when a pup ☐ More than once a month ☐ Every 1 to 2 months ☐ Every 3 months
☐ Every 4-6 months ☐ Annually ☐ Sporadically ☐ Never

3. **What flea treatment do you use for your dog(s)?**

☐ We don't use flea treatment
☐ Commercial dog product
☐ Non-medicated shampoo
☐ Wash with water / Swim
☐ Commercial non-dog product (e.g. cattle or sheep etc)

4. **Can your dog(s) kennel be moved?**

☐ Yes ☐ No

***If* Yes to (4); How often do you move your dog(s) kennel?**

☐ Less than once a year ☐ More than once a year ☐ Never

5. **Is your dogs run* raised off the ground?**

☐ Yes with solid floor ☐ Yes with slatted floor ☐ No

*** Run***; The confined area attached to the front of the kennel* or *the area the dog(s) can reach when chained to the kennel*

6. **How often do you clean your dog(s) kennel?**

☐ Less than once a year ☐ More than once a year ☐ Never

Record Number…………………..Date………………………

OWNERS NAME____

DOGS NAME ____

---

1. **How often do you feed your dog(s)?**

☐ Once a day ☐ More than once a day
☐ Only after exercise or work ☐ Other (*Please specify below)*

....

....

....

2. **What do you feed your dog(s)?** (Tick as many as apply)

   If possible can you please indicate the rough proportion of different feeds fed to your dog(s) below the tick box (i.e. 50% or half Commercial, 30% or one third Home kill, 20% or one fifth Scraps).

	☐ Commercial dog food	☐ Home kill	☐ Scraps	☐ Other *(Please specify below)*
**Proportion of Total;**				

....

....

3. ***If*** **you feed Home kill meat, offal or birth tissue to your dog(s) do you treat it first (i.e. freeze or cook it)?** *(If you don't feed Home kill go straight to Question 10.)*

☐ Yes ☐ No ☐ Sometimes

If **Yes or Sometimes to (8); How is the meat treated?** (Tick as many as apply)

☐ Frozen for greater than 7 days ☐ Frozen for less than 7 days
☐ Boiled or cooked for greater than 1 hour ☐ Boiled or cooked for less than 1 hour
☐ Other (*Please specify below)*

....

....

....

4. **How often does your dog(s) receive veterinary attention?**

☐ Only when vaccinated ☐ Frequently ☐ Occasionally ☐ Never

:ND OF SECTION 2

**** Please leave the following sections blank *****

Record Number…………………..Date……………………..
